# Efficacy and safety of dopamine agonists in traumatic brain injury: a systematic review of randomized controlled trials

**DOI:** 10.1186/cc9731

**Published:** 2011-03-11

**Authors:** AJ Frenette, S Kanji, L Rees, DR Williamson, MM Perreault, AF Turgeon, F Bernard, DA Fergusson

**Affiliations:** 1 Hôpital du Sacré-Coeur de Montréal, Canada; 2 Ottawa Hospital, Ottawa, Canada; 3 Montreal General Hospital, Montreal, Canada; 4 Hôpital Enfant-Jésus de Québec, Canada; 5 Ontario Health Research Institute, Ottawa

## Introduction

In the ICU, dopamine agonists (DA) have been used in TBI patients to augment or accelerate cognitive recovery and rehabilitation. However, the efficacy and safety of DA in this population is not well established.

## Methods

We conducted a systematic review of randomized controlled trials (RCT) examining the clinical efficacy and safety of DA in TBI. We searched MEDLINE, Embase and the Cochrane Central Register of Controlled studies up to June 2010. We sought trials comparing the effect of a DA with either placebo, standard treatment or another active comparator. We included trials addressing efficacy using any outcome measure as a primary outcome and/or safety. There was no restriction for age, date, or language of publication. We excluded unpublished and animal trials. Sensitivity analyses were planned to evaluate the potential effect of timing of TBI, age, drugs and year of publication on efficacy.

## Results

Among the 790 citations identified, 20 RCTs evaluating methylphenidate, amantadine and bromocriptine were eligible. Significant heterogeneity pertaining to timing from injury to randomization, mechanism of trauma, severity of TBI and age was observed between and within trials and precluded from any pooling of data. Efficacy outcomes included mainly neuropsychological measures of cognitive functioning. A total of 76 different neuropsychological tests were used, but most of them (59%) only once. For the 12 tests used in more than one study, statistically positive results were reproduced three times. Only five studies systematically assessed safety using predefined objective measures or tools. No trend could be drawn from the analysis of efficacy and safety in any of the predefined categories of outcome. Important sources of bias in the studies were of major concern, including inappropriate use of cross-over design and under-reporting of randomization methods.

## Conclusions

We observed a variability of neuropsychological tests. This may reflect disagreement regarding clinical relevance of cognitive and behavioral outcomes and lack of a gold standard test for each domain. Considering the absence of consensus along with the high risk of bias in included trials, more research is warranted before DA can be recommended to improve cognitive recovery in critically ill TBI patients.

